# The Principal of Dynamic Contrast Enhanced MRI, the Method of Pharmacokinetic Analysis, and Its Application in the Head and Neck Region

**DOI:** 10.1155/2012/480659

**Published:** 2012-10-18

**Authors:** Toru Chikui, Makoto Obara, Arjan W. Simonetti, Masahiro Ohga, Shoichi Koga, Shintaro Kawano, Yoshio Matsuo, Takeshi Kamintani, Tomoko Shiraishi, Erina Kitamoto, Katsumasa Nakamura, Kazunori Yoshiura

**Affiliations:** ^1^Department of Oral and Maxillofacial Radiology, Faculty of Dental Science, Kyushu University, 3-1-1 Maidashi, Higashi-ku, Fukuoka 812-8582, Japan; ^2^Philips Electronics Japan, Ltd. 2-13-37 Konan, Minato-ku, Tokyo 108-85 07, Japan; ^3^Imaging Systems, Philips Healthcare, Best, The Netherlands; ^4^Radiology Center, Kyushu University Hospital, Kyushu University, Fukuoka 812-85 82, Japan; ^5^Section of Oral and Maxillofacial Oncology, Division of Maxillofacial Diagnostic and Surgical Sciences, Faculty of Dental Science, Kyushu University, Fukuoka, Japan; ^6^Department of Clinical Radiology, Graduate School of Medical Sciences, Kyushu University, Fukuoka, Japan; ^7^Section of Image Diagnosis, Department of Diagnostics and General Care, Fukuoka Dental College, Fukuoka 814-0193, Japan

## Abstract

Many researchers have established the utility of the dynamic contrast enhanced-magnetic resonance imaging (DCE-MRI) in the differential diagnosis in the head and neck region, especially in the salivary gland tumors. The subjective assessment of the pattern of the time-intensity curve (TIC) or the simple quantification of the TIC, such as the time to peak enhancement (*T*_peak_) and the wash-out ratio (WR), is commonly used. Although the semiquantitative evaluations described above have been widely applied, they do not provide information on the underlying pharmacokinetic analysis in tissue. The quantification of DCE-MRI is preferable; therefore, many compartment model analyses have been proposed. The Toft and Kermode (TK) model is one of the most popular compartment models, which provide information about the influx forward volume transfer constant from plasma into the extravascular-extracellular space (EES) and the fractional volume of EES per unit volume of tissue is used in many clinical studies. This paper will introduce the method of pharmacokinetic analysis and also describe the clinical application of this technique in the head and neck region.

## 1. Introduction

 The technique of dynamic contrast enhanced (DCE) magnetic resonance imaging (MRI), in which multiphase MRI scans are taken following the intravenous injection of a contrast agent, has been widely used in clinical practice. Many researchers have established the utility of the DCE-MRI in the differential diagnosis of salivary gland tumors [1–3]. DCE-MRI can successfully demonstrate the nature of a lymphoma and is helpful for making a differential diagnosis from other lesions[4]. Some researcher have also attempted to utilize DCE-MRI for lesions in the jaw bone[5–7]. The most conventional assessment using DCE-MRI may, therefore, be to use the characteristics of the time-intensity curve (TIC) regarding the regions of interest (ROIs), which are delineated by the observers. The subjective assessment of the pattern of the TIC or the simple quantification of the TIC, such as the time to peak enhancement (*T*_peak_) and the wash-out ratio (WR), is also commonly used. 

Although the semiquantitative evaluations described above have been widely applied, they do not provide information on the underlying pharmacokinetic nature in the tissue. Moreover, an analysis based on the signal intensity (SI) is predominantly affected by the scan parameters. Therefore, it is difficult to compare the results obtained at different institutes. Conversely, a pharmacokinetic analysis enables the quantification of contrast agent exchange between the intravascular and the interstitial space [8–11]. Pharmacokinetic analyses based on the two compartment model, which can provide information about the microvessel permeability and the extracellular space, have been widely applied. The contrast medium can leak out of the vasculature at variable rates; hence, the temporal resolution of low molecular weight contrast media needs to be on the order of 5–20 seconds. 

 There is an another technique, which is called *T*_2_* or susceptibility contrast enhanced (DSC) MRI. The contrast medium used for this technique is assumed to be predominantly confined to the vascular space, which thus provides the valuable information about perfusion, such as the blood volume (BV), blood flow (BF), and transit time; therefore, the required temporal resolution is on the order of 1-2 seconds. *T*_2_*-weighted sequences are used to monitor the passage of the contrast media, because of a transient darkening of the tissue during the first passage of the contrast media. However, there are a few reports on DSC-MRI usage outside the brain. Especially, the image quality of the *T*_2_*-weighted images is poor in the head and neck region, because of the pneumatic space and the presence of oral prostheses. Therefore, this study focused on DCE-MRI.

## 2. The Subjective and Semiquantitative Analysis of TICs

 The validity of DCE-MRI has been established for the diagnostic analysis of salivary gland tumors [1–3]. Many studies have so far evaluated the TICs by placing an ROI on the tumor and comparing *T*_peak_ and WR. The *T*_peak_ for pleomorphic adenomas is longer than that for both malignant tumors and Warthin's tumors. Moreover, the WR for Warthin's tumors is the highest among those three lesions. A rapid increase—rapid washout pattern—suggests a high possibility of Warthin's tumor. A persistent increase pattern suggests the benign nature of a tumor, suggesting a high possibility of pleomorphic adenoma. A plateau pattern with slow washout is characteristic of both a malignant tumor and pleomorphic adenoma. Yabuuchi et al. successfully demonstrated the validation of DCE-MRI (22 benign tumors, 11 malignant tumors) and evaluated the correlations between the *T*_peak_ and microvessel count and between WR and cellularity-stromal grade. The microvessel count is thought to represent tumor vascularity, and the *T*_peak_ is short if the microvessel count is high. The WR depends on the difference in the amount of contrast material within the tumor between the intravascular and extravascular phases. Therefore, a tumor with a high cellularity-stromal grade retains less contrast medium and has a high WR [1]. A subsequent study combined DCE-MRI with the diffusion-weighted image and improved the diagnostic power for tumors with a plateau pattern [2].

 Many salivary gland tumors are composed of distinctive tissues, including proliferating tumor cells, myxomatous tissues, necrotic tissues, and cysts. Therefore, analyzing a large ROI may result in spurious results. A pixel-to-pixel evaluation of the TIC might enable effective and detailed characterization of the histological features. Eida et al. performed a pixel-to-pixel evaluation, in which each of the obtained TICs was automatically classified into four types on the basis of *T*_peak_ and WR into four types; type A (gradual enhancement), type B (rapid enhancement and low washout), type C (rapid enhancement and high washout), and type D (flat). Pleomorphic adenoma shows predominantly areas with type A. Warthin's tumor shows predominantly areas with type C; however, it also includes areas of type D, which corresponded to the microcysts. A malignant tumor shows areas with type A and type B. Therefore, a scattered type B area suggests the high possibility of malignancy [12, 13]. Their coworkers successively applied this method to other types of head and neck tumors [14, 15]. 

 DCE-MRI can successfully demonstrate the nature of a lymphoma and it is helpful for the differential diagnosis from another type of lesion. Some researchers applied DCE-MRI for evaluating lesions in the jaw bone [5–7]. Asaumi et al. demonstrated that DCE-MRI features of odontogenic myxomas are different from those of ameloblastomas and that a very slow gradual increase of signal intensity (SI) is characteristic of odontogenic myxomas [5]. However, ameloblastomas showed no difference in TIC pattern between histopathological types (plexiform, follicular, mixed desmoplastic, and unicystic type) [6].

 Although semiquantitative evaluations have been widely applied, they cannot provide sufficient information to make an underlying pharmacokinetic analysis of tissue. The quantification of DCE-MRI is preferable to predict tumor response to anticancer therapy and monitoring the tumor response to the therapy. Therefore, this study will introduce the method of pharmacokinetic analysis and also describe the clinical application of this technique.

## 3. The Pharmacokinetic Analysis of DCE-MRI

 There are many kinetic models; however, the Toft and Kermode (TK) model are usually used in clinical studies [8–10]. This model provide information about the influx forward volume transfer constant from plasma into the extravascular-extracellular space (EES) and fractional volume of EES per unit volume of tissue. A more elaborate pharmacokinetic model had been proposed to estimate additional characteristic parameters like BF, BV, and so on. For example, an adiabatic approximation of the tissue homogeneity model provides additional information. Therefore, it enables the permeability surface-area product to be measured separately from tissue perfusion [16–18]. However, the elaborated model is not yet supported by sufficient evidence and it is difficult to carry out in many institutes. Therefore, the following section introduces the theory of the two simple models, namely, the TK model [8–10] and Brix model [19, 20].

## 4. Theory 1: Toft and Kermode Model (TK Model) [8–10]

**Figure 1:**
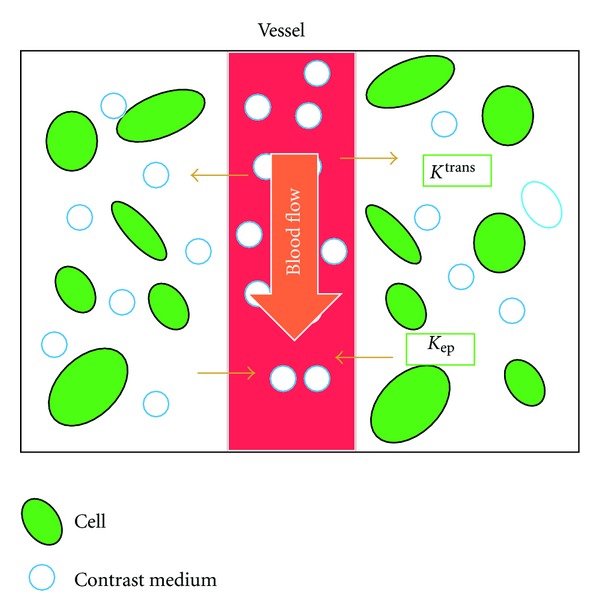
Schematic illustration of the TK model. The model assumes equilibrium of the contrast media between the plasma and the extravascular-extracellular space (EES) and the isodirectional permeability and *K* ^trans⁡^: influx forward volume transfer constant (into EES from plasma), *v*_*e*_: fractional volume of EES per unit volume of tissue, *K*_ep_: the efflux rate constant from the EES to plasma (*K*_ep_ = *K* ^trans⁡^/*v*_*e*_).

 The TK model is one of the popular compartment models, which assumes the equilibrium of contrast media between the plasma and the EES and the isodirectional permeability (Figure 1), therefore, the equilibrium is described by

(1) $$\begin{array}{c} \frac{dC_{t}}{dt}=K^{\mathrm{trans}}\left(C_{p}-\frac{C_{t}}{v_{e}}\right), \end{array}$$

where *t* is the time, *C*_*t*_ is the concentration of contrast media (CM) in tissue, *C*_*p*_ is the concentration of CM in plasma, *K* ^trans⁡^ is the influx forward volume transfer constant (into EES from plasma), and *v*_*e*_ is the fractional volume of EES per unit volume of tissue (Figure 1).

 The original TK model (one-compartment, two-parameter model) assumes the concentration of the CM is derived from the EES components and the plasma component is negligible:

(2) $$\begin{array}{c} C_{t}\left(t\right)=K^{\mathrm{trans}}\int_{0}^{t}C_{p}\left(t^{\prime }\right)\exp\left\{\left(-\frac{K^{\mathrm{trans}}\left(t-t^{\prime }\right)}{v_{e}}\right)\right\}dt^{\prime }. \end{array}$$

The modified TK model (two-compartment, three-parameter model) assumes the concentration of the CM is derived from the EES and plasma

(3) Ct(t)=Ktrans⁡∫0tCp(t′)exp⁡⁡{(−Ktrans⁡(t−t′)ve)}dt′+vpCp(t),

where *v*_*e*_ is the fractional volume of plasma per unit volume of tissue.

 Substituting *C*_*t*_ and *C*_*p*_ into either (2) or (3) allows the variables (*K* ^trans⁡^, *v*_*e*_, and *v*_*p*_) to be estimated. The pharmacokinetic analysis requires the *C*_*t*_; however, the relationship between the signal intensity (SI) and the concentration of the CM is not linear; therefore, conversion of the SI into the concentration of CM is needed.

 The key concept is that the increase in the relaxation rate is linearly related to the concentration in tissue:

(4) $$\begin{array}{c} \frac{1}{T_{1}}=\frac{1}{T_{10}}+R_{1}C, \end{array}$$

where *T*_10_ is *T*_1_ relaxation time before injection of CM, *T*_1_ is *T*_1_ relaxation times during the dynamic sequence (during and after injection of CM), *R*_1_ is a constant (relaxation rate) determined for each CM, and *C* is the concentration of CM during the dynamic sequence. 

 The first step for the estimation of *C* is to make an accurate *T*_10_ map. Most clinical studies using DCE-MRI include a precontrast *T*_1_-weighted image (3D-transverse-spoiled gradient echo sequence) with different flip angles to obtain a *T*_10_ map [21, 22]. This is followed by the dynamic contrast-enhanced series using the same sequence but with an FA, which is usually equal to the highest value in the pre-contrast image. The signal intensity of a transverse-spoiled gradient echo sequence is given by

(5) $$\begin{array}{c} {\text{SI}}_{0}=M\sin\alpha \frac{1-\exp\left(-\text{TR}/T_{10}\right)}{1-\cos\alpha \exp\left(-\text{TR}/T_{10}\right)}, \end{array}$$

where *M* is the proton density, *α* is the flip angle, TR is the repetition time. The pre-contrast data obtained with different flip angles substitute into (5) to yield the *T*_10_ map. 

 However, pulse sequences using a train of read-out pulse (e.g., variable flip angles) are particularly susceptible to the errors in the flip angles. Alternatively, magnetization prepared sequence-like inversion recovery and saturation recovery are less affected by the B_1_ nonuniformity. The Look-Locker (LL) sequence employs multiple radiofrequency pulses during the magnetization recovery to sample several time points and thereby track the recovery of magnetization, which have been applied for fast *T*_1_ mapping [23, 24]. This technique has also been successfully applied to the orofacial region [25]. 

 The *T*_10_ map is obtained first, then the *T*_1_ map during the dynamic contrast-enhanced series can be defined as

(6) $$\begin{array}{c} \frac{\text{SI}}{{\text{SI}}_{0}}=\frac{1-\exp\left(-\text{TR}/T_{1}\right)}{1-\cos\alpha \exp\left(-\text{TR}/T_{1}\right)}\frac{1-\cos\alpha \exp\left(-\text{TR}/T_{10}\right)}{1-\exp\left(-\text{TR}/T_{10}\right)}. \end{array}$$

**Figure 2:**
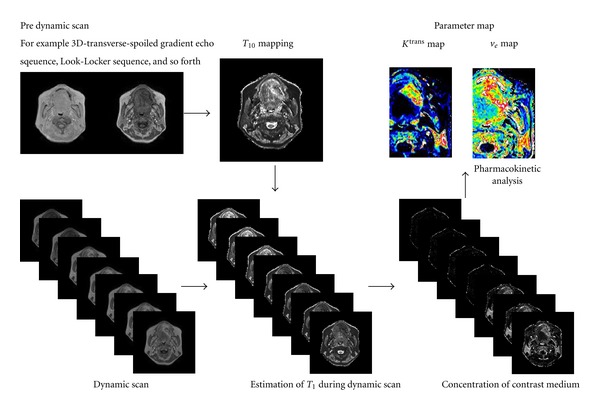
Diagram of the pharmacokinetic analysis.

The concentration of CM is estimated by the substitution of *T*_1_ and *T*_10_ obtained by (5) and (6) into (4) (Figure 2). 

The estimation of *C*_*t*_ (concentration of CM in tissue) was described above. The concentration of CM in the total blood (*C*_*b*_ = (1 − hematocrit) · *C*_*p*_) can be estimated by the same theory, if the region of interest (ROI) is placed in an artery; however, this estimation is quite inaccurate because of the high arterial CM concentration and the inflow effects. The high speed of the main artery and the vessel orientation, which is almost vertical to the axial DCE-MRI, makes it difficult to estimate an accurate *C*_*b*_, especially in the head and neck region. Alternatively, an empirical or population-derived input function describing a biexponential form is commonly used in this area [26–28]. 

**Figure 3:**
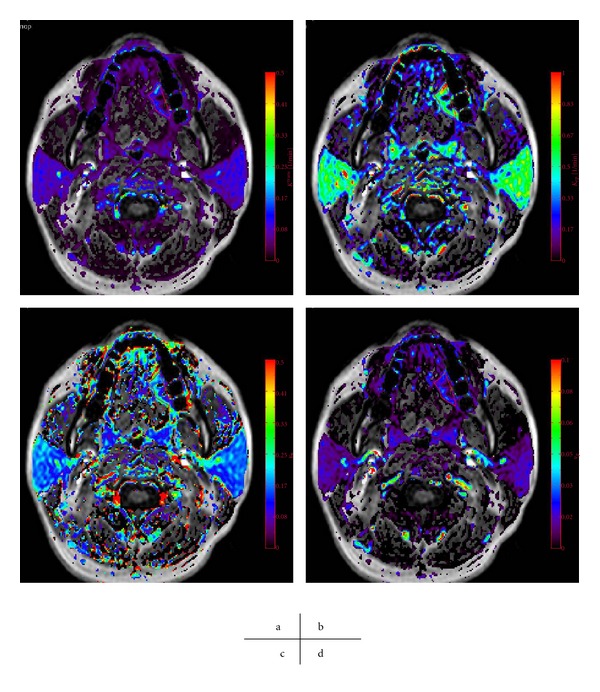
The parameter map of patients with left tongue cancer obtained by a TK model analysis. *K* ^trans⁡^ (a), *K*_ep_ (b), *v*_*e*_ (c), and *v*_*p*_ (d) map.

Substituting *C*_*t*_ and *C*_*p*_ into either (2) or (3) allows the parameter map (*K* ^trans⁡^, *v*_*e*_, and *v*_*p*_ map) to be estimated (Figure 3).

## 5. Theory 2: The Brix Model [19, 20]

**Figure 4:**
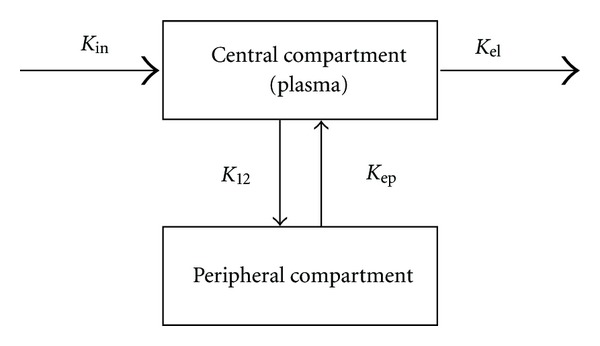
Schematic illustration of the Brix model. The model is a linear two-compartment open model, where the peripheral compartment has only negligible effects on the central compartment. *K*_ep_: the exchange rate constant from the EES to plasma, *K*_el_: the elimination constant of the CM from the central compartment, and AH: the amplitude scaling constant.

 The Brix model is a linear two-compartment open model, where the peripheral compartment has only negligible effects on the central compartment (Figure 4). The model was first applied to DCE-MRI with slow infusion. The *C*_*t*_ data were fitted to the Brix pharmacokinetic model as shown in the following equation:

(7) Ct=−AHKep−Kel[exp⁡⁡{(−Kep(t−TA))}       −exp⁡{(−Kel(t−TA))}],

where TA is the time of the arrival of the CM, *K*_el_ is the elimination constant of the CM from the central compartment, *K*_ep_ is the exchange rate constant from the EES to plasma, and AH is the amplitude scaling constant. AH is predominantly affected by the ratio of EES, although various indices like the properties of tissue (*T*_10_, *T*_20_, *K*_12_, ratio of EES, etc.), scan parameters (TR, TE), and infusion rates [19, 20]. 

The original Brix model assumed that the signal-time data (SI) were first normalized (SI_0_) and then converted to the concentration-time data (*C*_*t*_) based on the following relationship:

(8) $$\begin{array}{c} C_{t}=\delta \frac{{\text{SI}}_{t}-{\text{SI}}_{0}}{{\text{SI}}_{0}}, \end{array}$$

where SI_0_ is the baseline signal intensity before the injection of contrast medium and *δ* is a constant of proportionality. *C*_*t*_ could be approximated by (8) in a low concentration of CM and short TR/TE, although (8) is not strictly correct. It does not need *T*_10_ mapping or arterial input function measurements and therefore, it has been applied to clinical study as a simple quantitative method. However, the influx forward volume transfer constant into EES from plasma (*K* ^trans⁡^), which is an important parameter in the perfusion study, cannot be obtained, and this is considered to be a major drawback of this analysis.

## 6. Clinical Application of Pharmacokinetic Analysis

 Studies have demonstrated the utility of a pharmacokinetic analysis in the differential diagnosis of head and neck lesions. Lee et al. applied a pharmacokinetic analysis to 63 patients with 26 undifferentiated (UD) carcinomas, 28 squamous cell carcinoma (SCC), and 8 lymphomas. They demonstrated significant differences in the *K* ^trans⁡^ between UD and SCC and between UD and lymphoma. They suggested that their *K* ^trans⁡^ results appear to correlate with the expression of vascular endothelial growth factor (VEGF). The *v*_*e*_ of the lymphoma was the smallest among three types of tumor; however, the differences were not significant [29]. 

 Roberts compared parotid gland microvascular characteristics in patients with Sjögren's syndrome (*n* = 21) with those in healthy volunteers (*n* = 11). They demonstrated that the patients with Sjögren's syndrome had highly significant differences in both the *K* ^trans⁡^ and *v*_*e*_. Gland heterogeneity was significantly greater in the patients with Sjögren's syndrome [30].

 Lee et al. also evaluated radiation injury of the parotid gland during treatment for head and neck cancer (*n* = 21). DCE-MRI was performed before and 3 months after radiotherapy. The mean radiation dose was 47.1 ± 6.6 Gy and all the patients received concurrent chemotherapy. Three parameters (*K* ^trans⁡^, *v*_*e*_, and *v*_*p*_) were correlated with the dose of radiation delivered to the parotid gland and the degree of radiation-induced parotid atrophy [31].

Pharmacokinetic analyses have been widely applied to the pretreatment prediction of therapeutic efficacy and monitoring the tumor response to anticancer therapies. Several studies demonstrated that chemoradiotherapy (CRT) is more effective against tumors with a higher pretreatment *K* ^trans⁡^ than those with a lower *K* ^trans⁡^ and suggested that the elevated blood flow and permeable vasculature had higher oxygenation levels, thus resulting in better access to the chemotherapeutic drug and better radiosensitivity. Agrawal et al. performed DCE-MRI for twenty-one patients with advanced NHC, treated by concurrent CRT. All patients received a radical dose up to a dose of 70 Gy of conventionally fractionated radiotherapy (RT) along with concurrent weekly cisplatin. They found that the values of both BV and BF were higher than in complete responders in comparison to partial responders [32]. Kim et al. enrolled 33 HNSCC patients treated by neoadjuvant CRT. Treatment included accelerated RT with 220cGy per fraction for a total dose of 70.4 Gy. They demonstrated that the average pretreatment *K* ^trans⁡^ of the CR group was significantly higher than that of the PR group [33].

Studies have shown that staining for pimonidazole, an exogenous marker of hypoxia, is significantly associated with *K* ^trans⁡^ and *K*_ep_. Many HNSCC studies suggest a high pre-treatment permeability (*K* ^trans⁡^) is linked to favorable treatment outcome [34, 35].

 Many researchers demonstrated the utility of DCE-MRI for monitoring the tumor response to anticancer therapy in various tissues (breast, bladder, bone, etc.). A decrease of the *K* ^trans⁡^ suggests a good response to chemotherapy; however, an increase or no change suggests a poor response to chemotherapy. 

 Some HNSCC studies found that a large reduction of the permeability (*K* ^trans⁡^) is also linked to better response to CRT [36, 37]. However, other studies demonstrated conflicting results, where an increase of permeability (*K* ^trans⁡^) or blood volume (BV) after CRT suggests a favorable outcome. Cao et al. performed the quantification of blood volume and blood flow based on DCE-MRI taken before therapy and 2 weeks after the initiation of CRT (*n* = 14). They evaluated the local and regional control and concluded that an increase of BV and BF suggested the good outcome [38]. 

**Figure 5:**
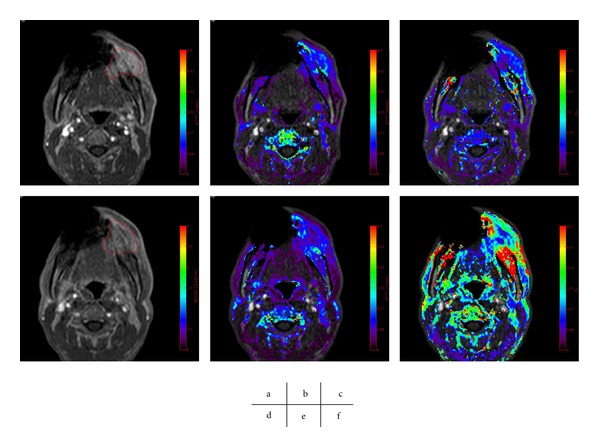
A poor tumor response to CRT, with an Ohboshi and Shimosato classification of I. The analyses were performed using a proprietary software program (PRIDE software, Philips Healthcare, Eindhoven, The Netherlands). Pre-CRT (a–c), Gd-enhanced *T*_1_ WI (a), *K* ^trans⁡^ map (b), *v*_*e*_ map (c), Post-CRT (d–f), Gd-enhanced *T*_1_ WI (d), *K* ^trans⁡^ map (e), *v*_*e*_ map (f). The tumor ROIs are delineated in red (a, d). The pre-CRT *K* ^trans⁡^ was 0.13 and *K* ^trans⁡^ decreased after CRT with an average of 0.10. *v*_*e*_ decrease from 0.28 to 0.23.

**Figure 6:**
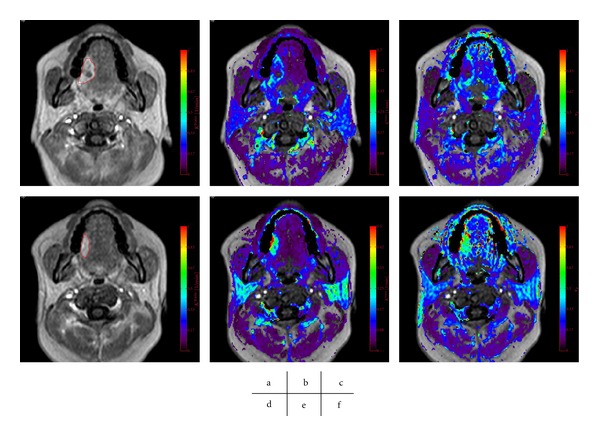
A good tumor response to CRT, with an Ohboshi and Shimosato classification of III. The analyses were performed using a proprietary software program (PRIDE software, Philips Healthcare, Eindhoven, The Netherlands). Pre-CRT (a–c), Gd enhanced *T*_1_ WI (a), *K* ^trans⁡^ map (b), *v*_*e*_ map (c), Post-CRT (d–f), Gd enhanced *T*_1_ WI (d), *K* ^trans⁡^ map (e), *v*_*e*_ map (f). The tumor ROIs are delineated in red (a, d). *K* ^trans⁡^ increase from 0.14 to 0.22 and *v*_*e*_ decrease from 0.28 to 0.52.

Chikui et al. [39] performed DCE-MRI before and after preoperative CRT (*n* = 29). The histological evaluation of the effects of CRT was performed according to Ohboshi and Shimosato's classification [40] in the excised specimen after the surgery. These criteria grade the tumor response from I (minimal change) to IV (complete disappearance of the tumor cells). Patients with grades IIb, III, and IV are considered responders (*n* = 19), while those with grades IIa and I are considered nonresponders (*n* = 10). The change of *K* ^trans⁡^ of the responders was significantly larger than from that of the nonresponders (*P* = 0.018) [39] (Figures 5 and 6).

 These conflicting results may be due to the type of therapy, the endpoint definition, the timing of the evaluation, and so on. The low radiation dose administered in this study caused an early vascular response, which typically involves a phase of vasodilatation and an increase in blood supply, similar to that observed during acute inflammation, although it is followed by the constriction of capillaries as the total dose increases. Mayer et al. reported the results of cervical carcinoma patients, who underwent DCE-MRIs at baseline and at week 2 and 5 of external beam RT. They demonstrated that the increased permeability at week 2 of the RT is associated with higher local control and overall survival rates. The tumor reoxygenation accompanied with the increased perfusion/permeability is attributed to the good tumor response [41].

 CRT causes a significant increase in the EES and moreover, the degree of tumor response is correlated with the increase in the EES. These were previous reports, in which the Brix model was used for patients with oral [42] and esophageal [43] carcinoma to compare the parameters between before and after CRT. AH was an important parameter for monitoring the tumor response and a large increase in AH shows a good tumor response to CRT. A TK analysis in the patients with oral SCC also demonstrated that a larger increase in *v*_*e*_ suggests a good response to the CRT (Figures 5 and 6). 

 The increase of apparent diffusion coefficient (ADC) in responders can be interpreted as a decrease in the cell density and an enlarged EES [44, 45]. Vandecaveye et al. reported the use of DWI for the detection of an early response to CRT treatment and demonstrated that an increase of ADC after treatment is indicative of a high possibility of local control [44]. The results of *v*_*e*_ obtained by DCE-MRI are consistent with the results of ADC obtained by diffusion-weighted MRI.

## 7. Conclusions

 The image assessment of DCE-MRI and a pharmacokinetic analysis together reveal the underlying tissue and tumor biology and thus provide additional information. The TK model analysis has established the validity of predicting the tumor response to the therapy and monitoring the tumor response. However, the parameters reported in the literature vary considerably; therefore, it is difficult to compare the parameters among research groups. Thus, further standardization of this analysis is required. An accurate *T*_10_ map and accurate arterial input function measurements are essential for the further development of a model analysis in the head and neck region.
